# Does protracted chemotherapy have an influence on the clinical outcomes in advanced epithelial ovarian cancer?

**DOI:** 10.1097/MD.0000000000029967

**Published:** 2022-08-12

**Authors:** Juhun Lee, Dae Gy Hong

**Affiliations:** a Department of Obstetrics and Gynecology, School of Medicine, Kyungpook National University, Kyungpook National University Chilgok Hospital, Daegu, Republic of Korea.

**Keywords:** adjuvant chemotherapy, ovarian cancer, protracted chemotherapy, survival outcomes

## Abstract

In epithelial ovarian cancer, first-line adjuvant chemotherapy is necessary, and patients sometimes require protraction; however, there are only a few recent studies to show its influence. In this study, we investigated whether the protraction of the total period of first-line chemotherapy has a negative influence on the survival outcomes.

Of the 101 patients we recruited from February 2011 to February 2021, 70 (69.3%) and 31 (30.7%) were classified into the not protracted and protracted groups, respectively. They underwent surgery and adjuvant chemotherapy for epithelial ovarian cancer. Protraction was defined as the overall duration of the first-line chemotherapy being more than 20 days longer than intended. Number of patients who underwent additional treatments such as bevacizumab or poly(adenosine diphosphate ribose) polymerase inhibitors or pembrolizumab was compared between both groups. Kaplan–Meier survival analysis and Cox regression analysis were used for survival outcomes.

There was no significant difference for additional treatments. The progression-free survival (PFS) in the total follow-up period in the protracted group was significantly shorter than that in the not protracted group (*P* = .037); however, the difference in the overall survival between the 2 groups was not significant (*P* = .223). For the PFS, the hazard ratio of protraction was 1.646 in the univariate analysis (95% confidence interval, 1.020–2.658; *P* = .041).

Excessive protraction of chemotherapy over 20 days or more can result in significantly shorter PFS within 5 years. A better therapeutic strategy is required for patients requiring protracted first-line chemotherapy in advanced epithelial ovarian cancer.

## 1. Introduction

Epithelial ovarian cancer is a very refractory disease due to its high risk for recurrence or progression. According to the American Cancer Society, there were approximately 21,000 new cases and 14,000 deaths due to this condition in 2021 as compared to the 282,000 new cases and 44,000 deaths due to breast cancer. For the advanced disease, the 5-year survival rate of ovarian cancer was 39% and 17% in stage III and stage IV, respectively, as compared to 86% and 28%, respectively, in breast cancer.^[1]^ Epithelial cancer accounts for more than 90% of all cases of ovarian cancer.^[2]^

For the treatment of epithelial ovarian cancer, surgery to reduce tumor burden as much as possible and adjuvant chemotherapy is necessary;^[3]^ even in the very early stage, chemotherapy is beneficial for prognosis.^[4]^

On chemotherapy, many gynecologic oncologists set 3 weeks between 2 administrations to manage the various toxicities; however, they also try not to delay too long. It is often necessary to postpone chemotherapy for patients’ poor general conditions or other medical interventions; thus, protraction occurs at a rate of approximately 23% to 58%.^[5–7]^ Previous studies have demonstrated the adverse effect on survival outcomes; however, the definition of protraction is still uncertain.

First-line adjuvant chemotherapy is vital in advanced epithelial ovarian cancer, and patients sometimes require protraction; however, there are only a few recent studies to show its influence. In this study, we investigated whether the protraction of the total period of first-line adjuvant chemotherapy has a negative influence on the survival outcomes.

## 2. Methods

**Figure 1.**
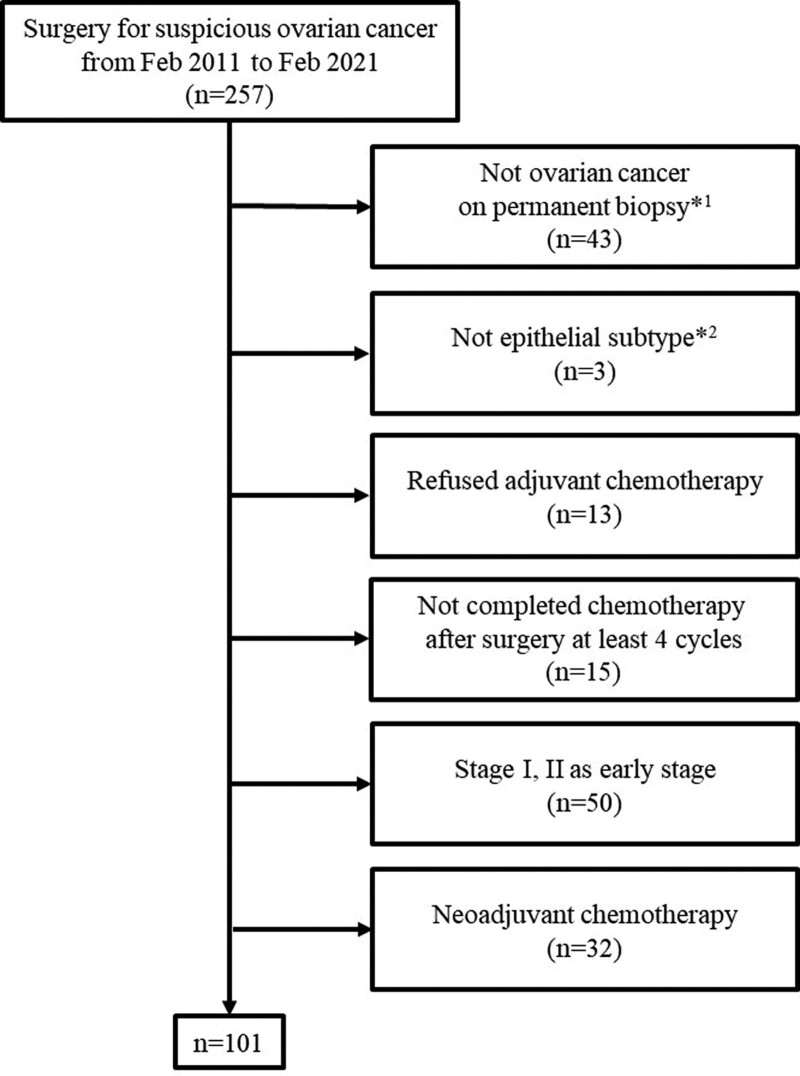
Flow diagram for patient selection. ^*1^Including benign, borderline malignancy, and other primary malignancies such as endometrial cancer or colon cancer. ^*2^Including sex-cord tumors and germ cell tumors).

From February 2011 to February 2021, 257 patients were retrospectively reviewed. They underwent surgery with or without first-line chemotherapy for ovarian tumor at the Kyungpook National University Chilgok Hospital. Forty-three patients were excluded due to the primary diagnosis on permanent biopsy, such as endometrial cancer and borderline tumor. Three participants whose histologic subtypes were not epithelial types were excluded as well. We excluded 13 patients who refused first-line adjuvant chemotherapy and 15 who did not complete at least 4 cycles of it.^[8]^ Fifty of the patients with early-stage (including stage I and stage II) ovarian cancer and 32 who were treated with neoadjuvant chemotherapy were excluded. Totally, 101 patients who were treated with the standard treatment were included in this study (Fig. 1). We evaluated the stage of each patient according to the International Federation of Gynecology and Obstetrics criteria.^[9]^ The institutional review board of our hospital approved this study (KNUCH 2021-12-031).

All surgical operations were performed by 4 experienced gynecologic oncologists at the hospital. The optimal debulking surgical operation included total hysterectomy, bilateral salpingo-oophorectomy, lymphadenectomy from both sides of the pelvis to the infrarenal level, omentectomy, and the resection of other metastatic lesions. We defined a suboptimal surgical operation as one in which the residual tumor was >1 cm.^[10]^

For the first-line adjuvant chemotherapy, a combination of taxane and carboplatin was used for all participants except only 1, a combination of taxane and cisplatin. One hundred one patients were divided into a protracted group and a nonprotracted group. Protraction was defined as the overall duration of first-line chemotherapy being >20 days longer than intended; for example, if 105 days is the intended period for 6 cycles of chemotherapy, patients who finished after 135 days were classified as the protracted group. The response to chemotherapy was confirmed by either computed tomography (CT), magnetic resonance imagining, or positron emission tomography/CT;^[11]^ such as complete or partial resolution, stable disease, progression, or recurrence.

Some patients underwent additional medical treatments according to their medical conditions. Those included hyperthermic intraperitoneal chemotherapy (HIPEC), bevacizumab, poly(adenosine diphosphate ribose) polymerase (PARP) inhibitors, and pembrolizumab. The HIPEC was performed during the primary debulking surgery. The bevacizumab group included patients who used it either together with first-line chemotherapy or as maintenance regimen after the chemotherapy. For PARP inhibitor or pembrolizumab, patient who used it at least once was included in each group (Table 1).

**Table 1:** Comparison of patients’ characteristics and clinical factors according to the protraction* of the first-line chemotherapy.

	**Nonprotracted**	**Protracted**	***P* value**
Number of patients (n)	70 (69.3%)	31 (30.7%)	
Age (yr)	54.99 ± 10.03	55.03 ± 11.95	.984
FIGO stage (n)			.056
III	67 (95.7%)	26 (83.9%)	
IV	3 (4.3%)	5 (16.1%)	
Histology (n)			.644
Serous	51 (72.9%)	24 (77.4%)	
Endometrioid	7 (10.0%)	2 (6.5%)	
Clear cell	3 (4.3%)	0 (0.0%)	
Mucinous	1 (1.4%)	0 (0.0%)	
Mixed epithelial, other	8 (11.4%)	5 (16.1%)	
Residual tumor (n)			1.000
Optimal surgery	51 (72.9%)	22 (71.0%)	
Suboptimal surgery	19 (27.1%)	9 (29.0%)	
Additional medical treatments (n)			
HIPEC	20 (28.6%)	11 (35.5%)	0.641
Bevacizumab	32 (45.7%)	14 (45.2%)	1.000
PARP† inhibitor	6 (8.6%)	3 (9.7%)	1.000
Pembrolizumab	3 (4.3%)	1 (3.2%)	1.000
For the first-line chemotherapy			
Interval to the first administration (d)	25.23 ± 8.76	22.29 ± 12.45	0.177
Regimen			1.000
Taxane and carboplatin (n)	69 (98.6%)	31 (100.0%)	
Others (n)	1 (1.4%)‡	0 (0.0%)	
Number of cycles (n)	6.71 ± 1.52	7.52 ± 1.50	0.016
Length of period (d)	125.17 ± 33.59	182.13 ± 39.37	0.000
Protracted days (d)§	5.17 ± 6.00	45.29 ± 23.64	0.000
Mean protracted days (d)∥	0.76 ± 0.92	6.35 ± 3.82	.000
Mean ratio of dose reduction (%)∥	4.74 ± 6.98	8.52 ± 8.51	.021
Total dose of administration (%)¶	637.79 ± 142.52	686.29 ± 142.52	.118
Serum level of CA-125 (U/mL)			
Preoperative	1326.711 ± 1879.80	1834.25 ± 2240.76	.241

Student *t* test and the chi-square test were used to compare both groups. The Kaplan–Meier survival analysis and Cox regression analysis were used for survival outcomes such as the overall survival (OS) and progression-free survival (PFS) and its hazard ratio (HR). All these statistical analyses were performed with SPSS (version 26; IBM Corp., Armonk, NY) and MedCalc (version 20.026; MedCalc Software Ltd, Belgium).

In accordance with the journal’s guidelines, we will provide our data for the reproducibility of this study in other centers if such is requested.

## 3. Results

Out of 101 patients, 70 (69.3%) and 31 (30.7%) were classified into the nonprotracted and protracted groups, respectively. No significant differences in age, stage, histology, residual tumor, and the preoperative serum level of cancer antigen 125 were found between both groups. For first-line chemotherapy, there were no significant differences in the regimen, interval to the first administration, and the total dose of the chemotherapy agent; however, there were significant differences in the number of cycles, the length of period, the mean ratio of dose reduction, and the number of protracted days. For additional medical treatments, no significant difference was found in the number of patients who underwent HIPEC or used bevacizumab, PARP inhibitor, or pembrolizumab (Table 1).

**Figure 2.**
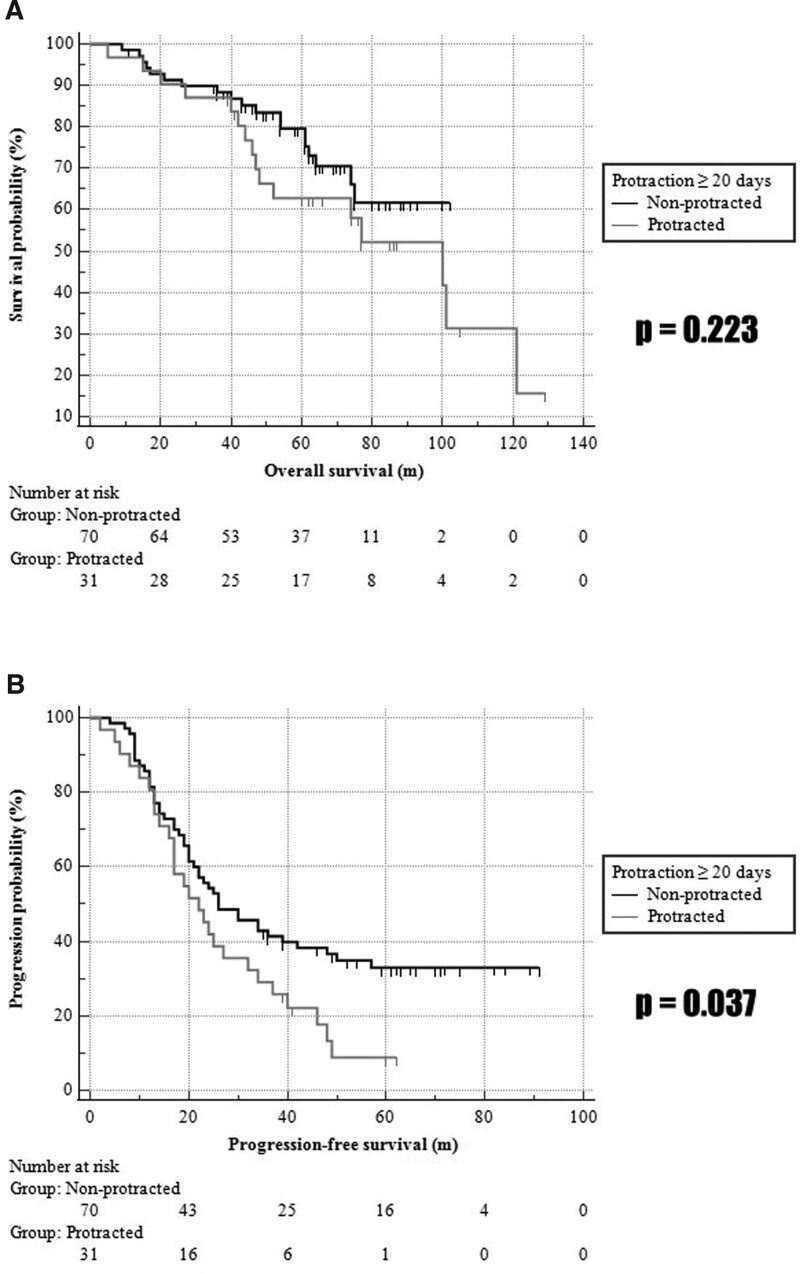
Comparison of survival curve and the number at risk for the overall survival and progression-free survival between the nonprotracted group and protracted* group in advanced epithelial ovarian cancer. *The overall duration of first-line chemotherapy was >20 d longer than intended.

The clinical outcomes were compared between both groups. The numbers of death for the follow-up period in the nonprotracted group and the protracted group were 7 (10.0%) and 11 (35.5%), respectively, whereas the numbers of survivals were 51 (72.9%) and 15 (48.4%), respectively. The death rate was significantly higher in the protracted group (*P* = .007). In 5 years, the OS rates in the nonprotracted group and the protracted group were 79.5% and 62.8%, respectively (*P* = .104), whereas the PFS rates were 32.9% and 8.8%, respectively (*P* = .003); in 3 years, the OS rates were 88.4% and 60.4% (*P* = .856) and the PFS rates were 41.4% and 29.0% (*P* = .219) in the nonprotracted group and the protracted group, respectively (Table 2). To evaluate the significances of the OS and PFS within the follow-up period, the Kaplan–Meier survival curve was analyzed; the number at risk was also shown (Fig. 2). The PFS of the protracted group was significantly shorter than that in the nonprotracted group (*P* = .037); however, the difference in OS was not statistically significant (*P* = .223). The HR for death and progression compared with protraction under 20 days were evaluated with multivariate and univariate Cox regression. For the OS, the HR of the protraction was 1.969 in multivariate analysis (95% confidence interval [CI], 0.450–8.618; *P* = .368) and 1.527 in univariate analysis (95% CI, 0.768–3.038; *P* = .227). For the PFS, the HR was 1.636 in multivariate analysis (95% CI, 0.621–4.309; *P* = .319) and 1.646 in univariate analysis (95% CI, 1.020–2.658; *P* = .041).

**Table 2:** Comparison of clinical outcomes according to the protraction* of adjuvant chemotherapy.

	**Nonprotracted (n** = **70**)	**Protracted (n** = **31**)	***P* value**
In follow-up period, n (%)			.007
Death	7 (10.0%)	11 (35.5%)	
Survival	51 (72.9%)	15 (48.4%)	
In 3 yr (%)			
Overall survival rate	88.4	87.1	.856
Progression-free survival rate	41.4	29.0	.219
In 5 yr (%)			
Overall survival rate	79.5	62.8	.104
Progression-free survival rate	32.9	8.8	.003

## 4. Discussion

The PFS was significantly shorter when the total period of first-line chemotherapy was protracted for over 20 days more than intended, whereas the shorter OS in the protracted group was not significant. The nonprotracted group had significantly shorter protraction and a lower ratio of dose reduction while on chemotherapy. It means that either their general conditions were better or their disease burden was lower than those in the protracted group; additionally, the proportion of stage IV disease was lower in the nonprotracted group. According to these, the improved performance status or less tumor burden could be related. Despite there was no significant difference in the total dose of chemotherapy agents and the additional medical treatments, the protracted group showed a shorter PFS. The proliferation of the residual cancer cells may not have been effectively suppressed due to the prolonged vacancy of chemotherapy agents; the mean lengths of protraction per cycle are presented in Table 1. Thus, a better therapeutic strategy is required for patients requiring protracted chemotherapy.

We conducted subgroup analyses to evaluate the influence of protraction in detail. The Kaplan–Meier survival curves for the OS and PFS according to 1, 5, 10, 15 days (*P* = .508, *P* = .788, *P* = .291, *P* = .296 [OS]; *P* = .148, *P* = .179, *P* = .784, *P* = .463 [PFS], respectively). We could not find statistical significance in these subgroup analyses.

We could search 2 recent studies showing the influence of chemotherapy protraction. In a Cochrane study, the authors classified patients who underwent dose reduction as well as protraction as the dose modification group; protraction was defined when the 8 cycles of chemotherapy were prolonged beyond 24 weeks.^[12]^ They showed significantly worse OS and PFS compared with the dose-unmodified group (HR = 1.26 [95% CI, 1.04–1.54], *P* = .021; HR = 1.43 [95% CI, 1.19–1.72], *P* < .001, respectively); however, the number of protractions was unclear in the total dose modification group (n = 229 [31%]). Another study demonstrated the negative effects of delayed completion of chemotherapy on survival. The authors evaluated the OS according to the number of cycles and the periods of adjuvant chemotherapy in the subgroup analysis.^[13]^ They found significantly shorter OS compared with the on-time complete subgroup in an advanced stage and protraction that lasted for over 4 weeks; however, there was no significant difference in the subgroup that had 1 to 4 weeks of protraction. Unlike them, we did not find a statistically significant difference in OS in subgroup analyses.

**Figure 3.**
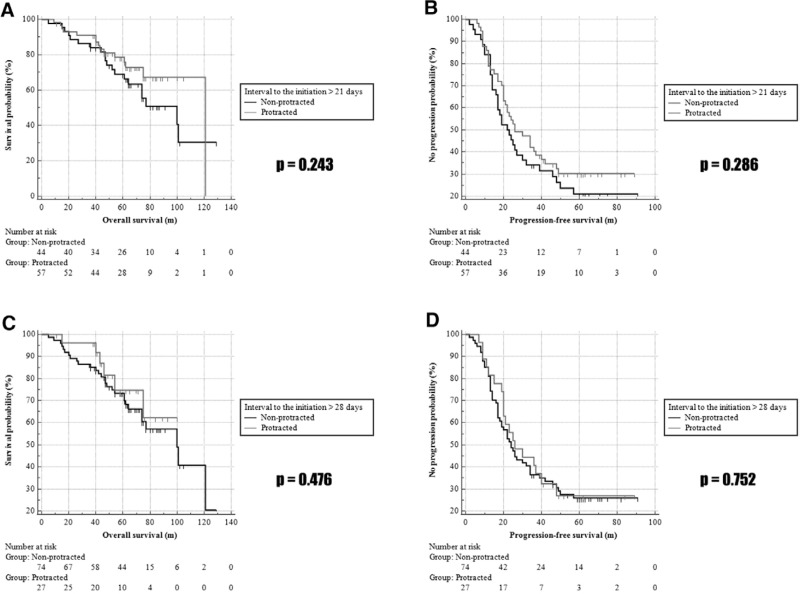
Kaplan–Meier curves for the OS and PFS according to the interval from primary debulking surgery to the first administration of chemo agents. OS = overall survival, PFS = progression-free survival.

Another subgroup analysis was performed to evaluate the relationship between the interval from primary debulking surgery to the first administration of chemotherapy agents and the OS and PFS (Fig. 3); the Kaplan–Meier survival curve was applied. When we classified 57 patients as the protraction over 21 days, both OS and PFS did not differ significantly (*P* = .243, *P* = .286) and also did not differ significantly over 28 days (N = 27; *P* = .476, *P* = .752, respectively).

We found a recent meta-analysis for the prolonged initiation of first-line chemotherapy. According to the authors, it was associated with a significant 22% risk decrease in the OS of ovarian cancer.^[14]^ In another observational study, the significantly shorter PFS was shown in the group that was protracted over 6 weeks; whereas the OS did not differ significantly.^[6]^ The results of our subgroup analyses were not comparable with those of previous studies; comparisons with our results from subgroup analyses were not appropriate because the meta-analysis included early-stage disease, and there were only 4 cases protracted over 6 weeks in our data.

Our study had 4 main limitations. First, it was a retrospective study in which data were collected at a single center. Second, we could compare between only 2 groups according to the protraction of 20 days due to limited sample size. Third, we did not evaluate the influence of the general performance status or tumor burden; thus, we could not demonstrate our inference for the PFS in survival curve analyses. Fourth, the effects of additional medical treatments such as bevacizumab, PARP inhibitor, pembrolizumab, and HIPEC on survival outcomes could not be evaluated appropriately. The bevacizumab could not be quantified because we used it with conventional chemotherapy together or as maintenance. The number of patients who used PARP inhibitor or pembrolizumab was too small to analyze. For the HIPEC, we only compared the number of patients between both groups and did not evaluate other factors in detail because of its controversial benefit on survival in advanced epithelial ovarian cancer.^[15]^

## 5. Conclusions

Excessive protraction of chemotherapy over 20 days or more can result in significantly shorter PFS within 5 years. A better therapeutic strategy is required for patients requiring protracted first-line chemotherapy in advanced epithelial ovarian cancer.

## Acknowledgments

We thank our colleagues Jong Mi Kim, Yoon Hee Lee, Gun Oh Chong, and Won Kee Lee who provided insight and expertise that greatly assisted the research.

## Author contributions

Conceptualization: Dae Gy Hong.

Data curation: Juhun Lee.

Formal analysis: Juhun Lee.

Investigation: Juhun Lee, Dae Gy Hong.

Methodology: Juhun Lee.

Project administration: Dae Gy Hong.

Supervision: Dae Gy Hong.

Validation: Dae Gy Hong.

Writing - original draft: Juhun Lee, Dae Gy Hong.

Writing - review & editing: Juhun Lee, Dae Gy Hong.

## Footnotes

* Of total period over 20 d than intention.

† Poly(adenosine diphosphate ribose) polymerase.

‡ A combination of taxane and cisplatin.

§ Total period of chemotherapy – (total number of cycles × 21 d).

∥ Per each cycle.

¶ Total dose of 6 cycles was defined as 600%.

* Protracted for over 20 d beyond the original intention.

## References

[1] American Cancer Society: American Cancer Society. Cancer Facts & Figures 2021. Atlanta: American Cancer Society. 2021:1–72.

[2] Sankaranarayanan R Ferlay J. Worldwide burden of gynaecological cancer: the size of the problem. Best Pract Res Clin Obstet Gynaecol. 2006;20:207–25. 16359925 10.1016/j.bpobgyn.2005.10.007

[3] Armstrong DK Alvarez RD Bakkum-Gamez JN. Ovarian cancer, version 2.2020, NCCN clinical practice guidelines in oncology. J Natl Compr Canc Netw. 2021;19:191–226. 33545690 10.6004/jnccn.2021.0007

[4] Lawrie TA Winter-Roach BA Heus P. Adjuvant (post-surgery) chemotherapy for early stage epithelial ovarian cancer. Cochrane Database Syst Rev. 2015;2015:CD004706. 10.1002/14651858.CD004706.pub5 PMC6457737 26676202

[5] Seagle BLL Butler SK Strohl AE. Chemotherapy delay after primary debulking surgery for ovarian cancer. Gynecol Oncol. 2017;144:260–5. 27908531 10.1016/j.ygyno.2016.11.022

[6] Singh S Guetzko M Resnick K. Preoperative predictors of delay in initiation of adjuvant chemotherapy in patients undergoing primary debulking surgery for ovarian cancer. Gynecol Oncol. 2016;143:241–5. 27615398 10.1016/j.ygyno.2016.09.004

[7] Sivakumaran T Mileshkin L Grant P. Evaluating the impact of dose reductions and delays on progression-free survival in women with ovarian cancer treated with either three-weekly or dose-dense carboplatin and paclitaxel regimens in the national prospective OPAL cohort study. Gynecol Oncol. 2020;158:47–53. 32381362 10.1016/j.ygyno.2020.04.706

[8] Bell J Brady MF Young RC. Randomized phase III trial of three versus six cycles of adjuvant carboplatin and paclitaxel in early stage epithelial ovarian carcinoma: a gynecologic oncology group study. Gynecol Oncol. 2006;102:432–9. 16860852 10.1016/j.ygyno.2006.06.013

[9] Prat J. Abridged republication of FIGO’s staging classification for cancer of the ovary, fallopian tube, and peritoneum. Cancer. 2015;121:3452–4. 26110780 10.1002/cncr.29524

[10] Salani R Axtell A Gerardi M. Limited utility of conventional criteria for predicting unresectable disease in patients with advanced stage epithelial ovarian cancer. Gynecol Oncol. 2008;108:271–5. 18164380 10.1016/j.ygyno.2007.11.004

[11] Eisenhauer EA Therasse P Bogaerts J. New response evaluation criteria in solid tumours: revised RECIST guideline (version 1.1). Eur J Cancer. 2009;45:228–47. 19097774 10.1016/j.ejca.2008.10.026

[12] Olawaiye AB Java JJ Krivak TC. Corrigendum to “Does adjuvant chemotherapy dose modification have an impact on the outcome of patients diagnosed with advanced stage ovarian cancer? An NRG Oncology/Gynecologic Oncology Group study” (Gynecologic Oncology (2018) 151(1) (18–23), (S0090825818310850)). Gynecol Oncol. 2019;152:220. 30135020 10.1016/j.ygyno.2018.07.021 PMC6151871

[13] Starbuck KD Szender JB Duncan WD. Prognostic impact of adjuvant chemotherapy treatment intensity for ovarian cancer. PLoS One. 2018;13:e02069131–12. 10.1371/journal.pone.0206913 PMC6231633 30418985

[14] Liu Y Zhang T Wu Q. Relationship between initiation time of adjuvant chemotherapy and survival in ovarian cancer patients: a dose-response meta-analysis of cohort studies. Sci Rep. 2017;7:1–8. 28842667 10.1038/s41598-017-10197-1 PMC5572704

[15] Lim MC Chang SJ Park B. Survival after hyperthermic intraperitoneal chemotherapy and primary or interval cytoreductive surgery in ovarian cancer: a randomized clinical trial. JAMA Surg. 2022;157:374–83. 35262624 10.1001/jamasurg.2022.0143 PMC8908225

